# First report of Matryoshka RNA virus in an African-European migrant bird

**DOI:** 10.1371/journal.pone.0319395

**Published:** 2025-03-04

**Authors:** Mélanie Duc, Carlos Esperanza, Carolina Romeiro Fernandes Chagas, Tatjana Iezhova, Ravinder N. M. Sehgal, Gediminas Valkiūnas

**Affiliations:** 1 P.B. Šivickis Laboratory of Parasitology, Nature Research Centre, Vilnius, Lithuania; 2 College of Biological Sciences, University of California, Davis, Davis, California, United States of America; 3 Department of Biology, San Francisco State University, San Francisco, California, United States of America; Universidade Federal de Minas Gerais, BRAZIL

## Abstract

Viruses are diverse biological entities found virtually in all environments on Earth. Their association with parasitic protozoans was shown in the late 1980’s, followed by evidence that these viruses can influence the treatment of infections as well as influence parasite virulence. Recently, Matryoshka RNA viruses (MaRNAV) were discovered in *Plasmodium vivax* infected patients in Malaysia, as well as in species of the closely related avian haemosporidian genera *Leucocytozoon* and *Haemoproteus* in Oceania and North America. However, they have not been reported in other continents so far. The aim of this study was thus to screen haemosporidian infected European birds (African migrants and residents) for the presence of MaRNAV. Whole blood samples from wild birds were collected in Lithuania in May 2023. Haemosporidian parasite infections were first assessed by microscopic examination and later confirmed via PCR. RNA was isolated and tested by Reverse Transcriptase (RT) PCR for the presence of MaRNAV. Of the 12 samples that were RT-PCR-positive, only one from a common whitethroat (*Curruca communis*) had a sequence with 63% similarity to MARNAV-2 found in *Leucocytozoon* infected birds from Oceania. Total RNA from this sample was sequenced, bioinformatically analyzed, and a new virus, MaRNAV-7, was identified. At the amino acid level, it is phylogenetically closely related to MaRNAV-2, MaRNAV-3 and MaRNAV-6 RdRp sequences, all found in *Leucocytozoon* infected birds. This is the first report of MaRNAV in an African-European haemosporidian infected bird, and a first step in understanding MaRNAV prevalence, distribution, and specificity. However, the effects that MaRNAV can have on the parasites, modulation of the host immune response and transmission rates remain unknown.

## Introduction

Viruses are the most abundant and diverse biological entities on Earth and are found in nearly every environment [1]. Metagenomic studies have shown that viruses are more diverse than their targets [2], which can include not only animals but also plants, fungi, bacteria and protozoans [1,2]. Since the late 1980’s, RNA viruses infecting protozoans (known as Parasitic Protozoan Viruses) have been reported in the human parasites *Giardia lamblia*, *Leishmania* spp.*, Trichomonas vaginalis, Cryptosporidium parvum*, and *Toxoplasma gondii* [3]. Recently, studies have shown that the presence of certain viruses can increase the risk of treatment failure in *Leishmania* infected individuals [4], or decrease the virulence in *G. lamblia* infections [5].

In 2019, a new virus, Matryoshka RNA viruses (MaRNAV), infecting parasitic protozoans was discovered. They are characterized by a bi-segmented genome consisting of an RNA-dependent RNA polymerase (RdRp) and a hypothetical protein sequence with two overlapping open reading frames (ORFs) with no known function [6]. The first was reported in humans and mosquitoes infected with *Plasmodium vivax* in eastern Malaysia and named MaRNAV-1. MaRNAV-2 was later found in several Australian birds infected with *Leucocytozoon* species [6], haemosporidian parasites closely related to *Plasmodium* and *Haemoproteus* pathogens [7]. New MaRNAV strains have since been discovered in American birds infected with the genera *Leucocytozoon* (MaRNAV-3 and MaRNAV-6) and *Haemoproteus* (MaRNAV-4 and MaRNAV-5) [8,9]. However, no records of MaRNAV have yet to be reported from other continents.

Noteworthy, avian haemosporidian parasites, the putative hosts of MaRNAV, have a worldwide distribution and are highly diverse, with almost 300 species described [10–12], and more than 5130 lineages (or haplotypes) reported (MalAvi, http://130.235.244.92/Malavi/ [13], accessed 2024-10-22). It is likely that other MaRNAV are to be found in wildlife. The aim of this study was to screen haemosporidian parasite infected European birds (African migrants and residents) for the presence of MaRNAV.

## Materials and methods

### Ethical statement

Animal procedures were approved by the Lithuanian Environmental Protection Agency, Vilnius (permit 2023-03-15 Nr. SR-197). All efforts were made to minimize bird handling and suffering.

### Sampling, DNA and RNA extractions, and PCR

Twenty-two bird species were sampled in May 2023 at the Ventės Ragas ornithological station, Lithuania (55º20’38.93” N, 21º11’34.05” E). Blood was collected to prepare blood smears, and to be stored in SET buffer (0.05M Tris, 0.15M NaCl, 0.5M EDTA, pH = 8.0). Blood smears were air-dried, fixed in absolute methanol, stained with a Giemsa solution, and screened using a microscope to determine infections with haemosporidian parasites (*Plasmodium*, *Haemoproteus* and/or *Leucocytozoon*) [14]. Blood was collected, stored in Trizol (Thermo Fischer Scientific, Vilnius, Lithuania), and immediately frozen in liquid nitrogen for selected individuals with single infections (only one haemosporidian parasite species), co-infections of haemosporidian species and non-infected individuals.

DNA from blood stored in SET buffer was extracted following an ammonium acetate protocol [15]. To confirm parasite infections and identify their lineages, PCRs were performed following established protocols using the primers HaemNFI/HaemNR3 for the first PCR and HaemF/HaemR2 and HaemFL/HaemR2L for the second PCR [16,17].

Single infections confirmed by microscopic examination were further processed for RNA extraction using an Invitrogen PureLink RNA mini kit with PureLink DNase I on-column treatment, followed by cDNA synthesis (SuperScript IV RT) (Thermo Fischer Scientific, Vilnius, Lithuania) using the manufacturer’s protocols. The presence of MaRNAV was tested by Reverse Transcriptase PCR (RT-PCR) using four different sets of primers (Table 1). The PCR temperature cycle profile started with an initial denaturation step at 94ºC for 5min, followed by 35 cycles of 94ºC for 30s, 55ºC for 30s and 72ºC for 30s, and a final extension at 72ºC for 7min. PCRs products were checked on a 2% Agarose gel for successful amplifications, which were prepared for sequencing and sent for Sanger bi-directional sequencing (Macrogen Europe, Amsterdam, The Netherlands).

**Table 1:** PCR primers used to screen for the presence of Matryoshka RNA viruses in haemosporidian parasite infected bird blood samples.

Primer	Primer sequence 5’-3’	Targeted MaRNAV	Reference
BW_Narnalike_FW1	CTGAAATTGATAARGAYGAAACTCC	MaRNAV2	[6]
BW_Narnalike_Rev1	CGTGGCATCCTTYAAATCTGATG	MaRNAV2	[6]
BW.Narna.Novel.3F	TCCATAAATGATGGGAGTAATCGC	MaRNAV2 second fragment	[6]
BW.Narna.Novel.2R	GATCTTGTATATACATAGATCCAATACAG	MaRNAV2 second fragment	[6]
MaRNAV4_F	ATTTATGAGTTCGGGGCCAG	MaRNAV4	[9]
MaRNAV4_R	TGAACCCATGACAAAGCCAT	MaRNAV4	[9]
MaRNAV5_F	TAGGAACGTGTAACCCCAGA	MaRNAV5	[9]
MaRNAV5_R	GAACACTTCCAACCGTGGTA	MaRNAV5	[9]

### RNA sequencing and bioinformatic analyses

One sample was positive when screening with the primers BW.Narna.Novel.3F/BW.Narna.Novel.2R and was sent for RNA sequencing (library TruSeq stranded total RNA with Ribo-Zero Gold and Sequencing NoveSeqX 150 bp Paired-end) to Macrogen Europe (Amsterdam, the Netherlands). Two files of about 1130-1140MB were received and analyzed bioinformatically to search for the virus. Briefly, the sequences were trimmed using Trimmomatic v0.40 [18], and transcriptomes were assembled by using Trinity *de novo* assembly software v2.10.0 [19]. All available sequences of RdRp and hypothetical protein of MaRNAV (as of May 2024) were exported from GenBank and used as a reference library for Diamond BLASTx v0.9.24 [20]. Hit sequences longer than 500 bp were subjected, one by one, to BLASTx and BLASTn in GenBank against the entire nr database to confirm if they matched only to the deposited MaRNAV. The longest open reading frame (ORF) of sequences confirmed to match a MaRNAV was then determined with NCBI ORF finder (https://www.ncbi.nlm.nih.gov/orffinder/), and its corresponding protein sequence and function were checked using both HHpred [21,22] and Protein Homology/analogy Recognition Engine v 2.0 (Phyre2) [23].

The analyses (Trimmomatic, Trinity, Diamond BLASTx) were repeated using the online server Galaxy (https://usegalaxy.org/), and the hits were checked in BLASTn, BLASTx, ORF finder, and HHpred and Phyre2. To compare and confirm the repeatability of the analyses and results, the obtained MaRNAV sequences from both servers were aligned, and a consensus sequence of the virus was created.

All records by searching “*Leucocytozoon*”, “*Haemoproteus*” and “Avian *Plasmodium*” from GenBank were exported and used as three libraries to BLASTn the RNA transcripts in search for the parasites.

### Phylogenetic analysis

A phylogeny was run using the protein sequence (RdRp) obtained from the longest ORF of the consensus. An alignment was created using MAFFT v7.490 [24,25] of RdRp sequences (MaRNAV and viruses following previous studies [6,8,9]) retrieved from GenBank, in Geneious Prime v.2024.0.7 (https://www.geneious.com). The best-fit model was determined as LG + I + G + F using MEGAX [26] and run in Geneious Prime using PhyML [27].

## Results

### Virus discovery

Although 44 samples were tested, just one sample yielded a PCR product amplified with the primers BW.Narna.Novel.3F and BW.Narna.Novel.2R. With this sample, only the forward sequence had 63% identity to MARNAV-2 hypothetical protein (QGV56803). This sample was then sequenced for total RNA. One transcript of more than 3000 bp in each analysis, with the longest ORF, matched MaRNAV-2 RdRp with > 75% identity (S1 Table), differed by 7 bp, and one amino acid. The obtained consensus sequence of these two transcripts (4972 bp) was identified as an RdRp (ORF =  3039nt/ 1012aa; Table 2) and named Matryoshka RNA Virus 7 (MaRNAV-7). A second transcript matching the hypothetical protein of MaRNAV-2 with 69% identity was found in both analyses (16 bp difference), with two overlapping ORFs (ORF1: 741nt/ 246aa; ORF2: 585nt/ 194aa) (Table 2; S1 Fig).

**Table 2:** Results of specific hits found for MaRNAV-7 and its hypothetical protein.

Name	MaRNAV-7	MaRNAV-7 hypothetical protein 1.1	MaRNAV-7 hypothetical protein 1.2
Length transcript (nt)	4972	1373	1367
Percentage identity to Matryoshka virus	76–82%	69%	69%
BLASTn	MaRNAV-2 RdRp	n/a	n/a
BLASTx	MaRNAV-2 RdRp	Hypothetical protein MaRNAV-2	Hypothetical protein MaRNAV-2
Longest ORF (nt/ aa)	3039/ 1012	741/ 246	741/ 246
HHphred results (e-value)	RdRp beta chain. 99.89% probability (3.3e–22)	Vesicle transport through interaction with t-SNAREs homolog. 64.53% probability (56)	Vesicle transport through interaction with t-SNAREs homolog. 64.23% probability (58)
Phyre2 results (percentage confidence)	DNA/RNA polymerases. 13% identity (83.5%)	hydrolase activator/protein transport. 28% identity (7.2%)	hydrolase activator/protein transport. 28% identity (7.2%)

### Host assignment

The MaRNAV-7 sequence was found in a common whitethroat (*Curruca communis*) individual positive by microscopy for parasites of the three genera, *Leucocytozoon* species (0.05% parasitemia), *Haemoproteus* sp. (only one gametocyte seen), and *Plasmodium* sp. (only three young trophozoites seen), and by PCR and sequencing with the cytb lineages lRECOB3 (*Leucocytozoon* sp.), hCWT2 (*Haemoproteus* sp.) and pTURDUS1 (*P. circumflexum*). Only *Leucocytozoon* (lRECOB3) was found in the RNA transcripts matching at least 478 bp with 100% identity.

**Fig 1:**
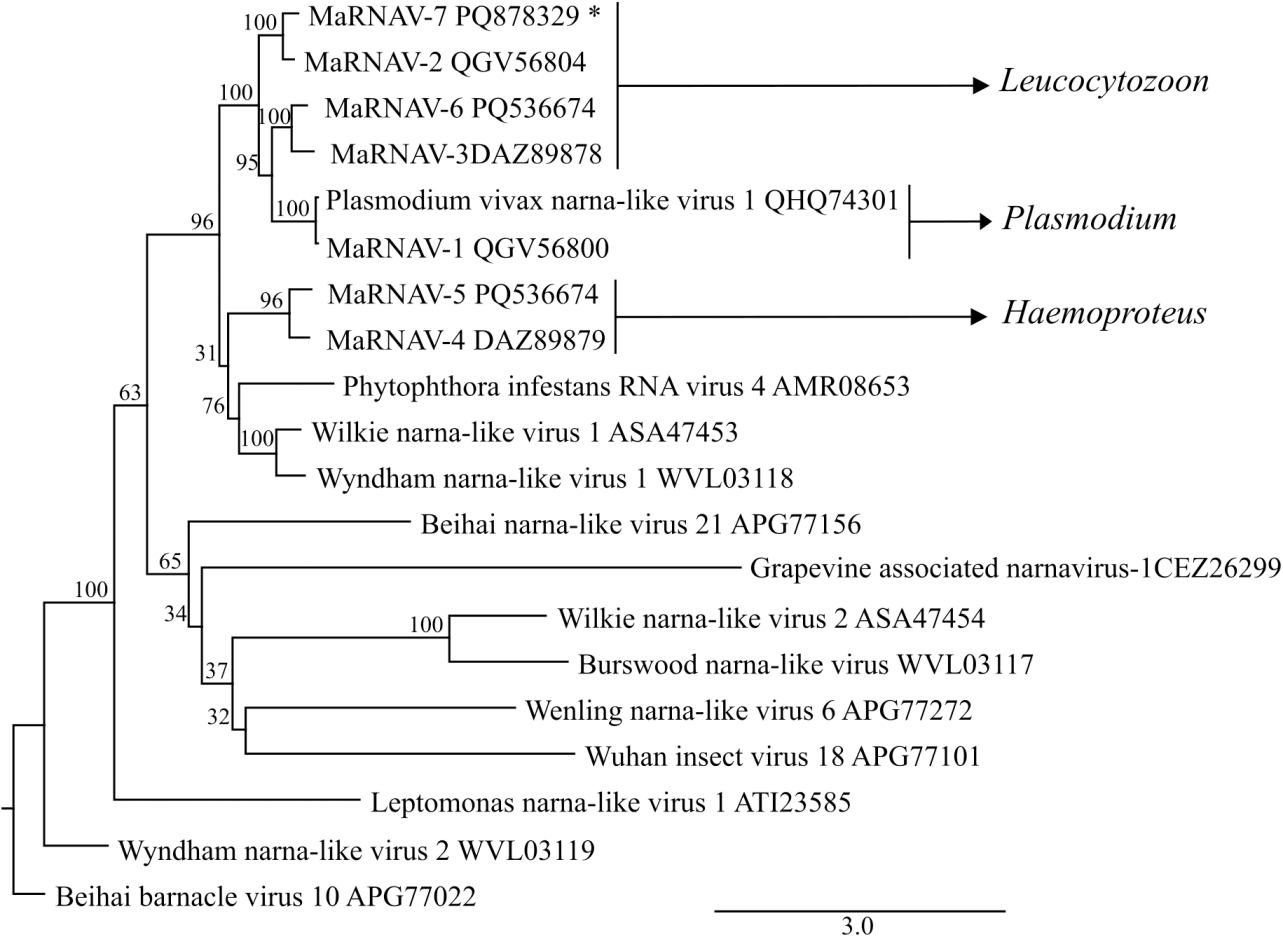
PhyML phylogeny of narna virus RdRp (RNA-dependent RNA polymerase) sequences. Matryoshka RNA viruses were found in birds infected with *Leucocytozoon* and *Haemoproteus* parasites, and in humans with *Plasmodium vivax*. The obtained sequence of this study gave a concordant result by clustering together with those of RNA virus obtained from other *Leucocytozoon* spp. infected samples. All bootstrap values are shown at the nodes. GenBank accession numbers are provided.

The phylogeny (Fig 1) showed the MaRNAV-7 sequence clustering closely with MaRNAV-2, MaRNAV-3 and MaRNAV-6 RdRp sequences (found in birds infected with *Leucocytozoon* sp.) and MaRNAV-1 (found in humans infected with *Plasmodium vivax*). MaRNAV-4 and MaRNAV-5 sequences from birds infected with *Haemoproteus* sp. clustered separately. Based on this information, we infer that MaRNAV-7 is a virus associated with *Leucocytozoon* parasites.

## Discussion

This study presents the first identification of MaRNAV in an African-European haemosporidian parasite infected common whitethroat. A homology-based search revealed transcripts encoding for RdRp with 38–81% similarity to previous MaRNAV RdRps (S2 Table), as well as additional genes encoding the hypothetical protein from MaRNAV-2. This new virus was named Matryoshka virus 7 (MaRNAV-7), following the discoveries of six other viruses in haemosporidian infected individuals [6,8,9]. The available data point out to MaRNAV-7 infecting a *Leucocytozoon* sp. (lineage lRECOB3) as (i) it is closely related to MaRNAV-2 found in Australian birds infected with *Leucocytozoon* parasites (Fig 1), (ii) the *Leucocytozoon* parasitemia was significantly higher than that of the other haemosporidians in the host, (iii) transcriptomes BLASTn resulted in hits to *Leucocytozoon* sequences, but no hits for *Haemoproteus* or *Plasmodium*, (iv) a second fragment (hypothetical protein) was also found in the sample, like in the other MaRNAV *Leucocytozoon* infected birds.

In terms of geographical distribution, MaRNAV have been reported in Oceania and North America, and now in an African-European bird [6,8,9]. This bird was mostly infected with *Leucocytozoon* sp. lRECOB3, which has been reported in Europe, Africa, and Asia in a total of 13 countries, and in 26 bird species, representing 12 bird families (MalAvi, accessed 2024-10-22). However, this is only one record of MaRNAV in the present study and in Europe, and it remains unknown how common MaRNAV are in the continent. The diagnosis of MaRNAV in avian hosts infected with haemosporidian parasites is challenging due to the high mutation rates of RNA viruses [28,29], which render conventional PCR protocols less efficient in diagnosis, if not for specific-MaRNAV strain primers [6,9]. Targeting bird species known to be infected with lRECOB3 might help to understand the prevalence of MaRNAV-7 in wildlife. Additionally, the recent finding of the same virus in different bird species, and even orders [9], points out the possibility of involvement of vectors in the infection process. The transmission of the virus from one parasite to another would happen in the vector, which would then transmit the associated parasites-virus to different bird species in the same locality. This would not be surprising, since vectors are in contact with a high diversity of hosts, bacteria, and viruses [30,31], and virus distribution could be correlated with the vector occurrence. SRA data analyses of *Anopheles* mosquitoes [6] showed the presence of MaRNAV-1 only when *P. vivax* infection was also present. However, no investigations have been carried out targeting MaRNAV in avian haemosporidian vectors which belong to the Simuliidae, Culicidae, Ceratopogonidae, and Hippoboscidae families.

Haemosporidian co-infections are commonly found in wild birds, rendering it difficult to properly assign the virus to its potential protozoan host. Six out of seven MaRNAV were found in birds infected with haemosporidians, and more are expected to be found, knowing the high diversity and geographical distribution of avian haemosporidians [10–13]. However, previous findings of MaRNAV did not confirm parasitemia, the presence of parasites in the blood, nor the lineages of the infection [6,8]. It is then difficult to compare different records aside from the parasite genus identification. In our study, *Leucocytozoon* was found with a higher parasitemia than *Plasmodium* and *Haemoproteus*, which might also explain the presence of only lRECOB3 in the RNA transcripts. However, it is too soon to draw any conclusions if MaRNAV provides any benefit to *Leucocytozoon* parasites when found in co-infections, and at which stage of the parasite development the virus is transmitted.

Interestingly, MaRNAV have yet to be found in avian *Plasmodium*, while it has been reported in humans with *Plasmodium vivax*, but not with *P. falciparum* or *P. knowlesi* [6]. One peculiarity of the *P. vivax* (and *P. ovale*) life cycle compared to other species infecting humans and birds, is the development of hypnozoites, the dormant stages persisting in the liver [32–34]. Based on current knowledge, avian haemosporidians do not develop hypnozoites, but other tissue stages (phanerozoites for *Plasmodium* spp., megalomeronts and meronts for *Leucocytozoon* and *Haemoproteus* [35]). It is possible that MaRNAV might be specific to species with certain tissue stages. However, the life cycle is not completely known for most of the avian haemosporidian species [35]. Among the *Plasmodium* species that develop hypnozoites stages are *Plasmodium cynomolgi* in monkeys and *P. berghei* in rodents for which SRA data were screened for MaRNAV [6]. The viruses were not found, however, most of the records came from experiments and cultures, which might impact the virus prevalence. Natural infections of *Plasmodium* species which develop hypnozoites should be investigated in different hosts to clarify this question.

Further studies targeting MaRNAV and how they are related to haemosporidian parasites life cycle are essential to understand not only their diversity and prevalence, but also their effect on host immunological response modulation, parasites virulence, parasitemia and transmission rates and host-parasite-vectors relationships.

## Supporting information

S1 Table Results of specific hits found for transcripts from analysis made in two servers. MaRNAV-7 sequences were aligned, and the resulting consensus was used for the analyses presented in the article and submitted to GenBank.
(PDF)

S2 Table Distance matrix of MaRNAV with percentage identity obtained at the nucleotide level (upper) and amino acids levels (lower). (PDF)

S1 Fig Nucleotide alignment and open reading frames (ORFs) predictions from the second segment of MaRNAV-7, hypothetical proteins. Nucleotide polymorphisms are indicated in black.
(TIFF)
