# Supplementary material for: First report of Matryoshka RNA virus in an African-European migrant bird
Source: PLoS One. 2025 Mar 4;20(3):e0319395. doi: 10.1371/journal.pone.0319395 (PMC11878896; doi:10.1371/journal.pone.0319395)
Supplement: S1 Table — MaRNAV-7 sequences were aligned, and the resulting consensus was used for the analyses presented in the article and submitted to GenBank. (PDF) [file pone.0319395.s001.pdf]

S1 Table. Results of specific hits found for transcripts from analysis made in two servers. MaRNAV-7 sequences were aligned, and the resulting consensus was used for the analyses presented in the article and submitted to GenBank.

**Sequence ID and server**	**MaRNAV-7 from server**	**MaRNAV-7 from Galaxy**	**MaRNAV-7 hypothetical protein 1.1 from server**	**MaRNAV-7 hypothetical protein 1.2 from Galaxy**
Length transcript (nt)	3654	3517	1373	1367
Percentage identity to Matryoshka RdRp	76 - 79%	75-82%	69%	69%
BLASTn	MaRNAV-2 RdRp	MaRNAV-2 RdRp	n/a	n/a
BLASTx	MaRNAV-2 RdRp	MaRNAV-2 RdRp	Hypothetical protein MaRNAV-2	Hypothetical protein MaRNAV-2
Longest ORF (nt / aa)	1950 / 649	3039 / 1012	741 / 246	741 / 246
HHphred results (e-value)	RdRp Beihai narna-like virus. 100% probability (1.2e-37)	RdRp beta chain. 99.89% probability (3.3e-22)	Vesicle transport through interaction with t-SNAREs homolog. 64.53% probability (56)	Vesicle transport through interaction with t-SNAREs homolog. 64.23% probability (58)
Phyre2 results (percentage confidence)	DNA/RNA polymerases. 13-18% identity (91-95%)	DNA/RNA polymerases. 13% identity (83.5%)	hydrolase activator/protein transport. 28% identity (7.2%)	hydrolase activator/protein transport. 28% identity (6.1%)
