# Supplementary material for: First report of Matryoshka RNA virus in an African-European migrant bird
Source: PLoS One. 2025 Mar 4;20(3):e0319395. doi: 10.1371/journal.pone.0319395 (PMC11878896; doi:10.1371/journal.pone.0319395)
Supplement: S2 Table — (PDF) [file pone.0319395.s002.pdf]

S2 Table. Distance matrix of MaRNAV with percentage identity obtained at the nucleotide level (upper) and amino acids levels (lower).

Segment I	MaRNAV-1	MaRNAV-2	MaRNAV-3	MaRNAV-4	MaRNAV-5	MaRNAV-6	MaRNAV-7
**MaRNAV-1**		53	55	29	28	54	51
**MaRNAV-2**	60		56	34	32	54	72
**MaRNAV-3**	58	61		31	30	66	39
**MaRNAV-4**	41	43	39		70	30	23
**MaRNAV-5**	35	44	39	71		29	32
**MaRNAV-6**	57	61	72	42	40		55
**MaRNAV-7**	54	80	53	38	37	58	
