# Supplementary figures and images for: First report of Matryoshka RNA virus in an African-European migrant bird
Source: PLoS One. 2025 Mar 4;20(3):e0319395. doi: 10.1371/journal.pone.0319395 (PMC11878896; doi:10.1371/journal.pone.0319395)

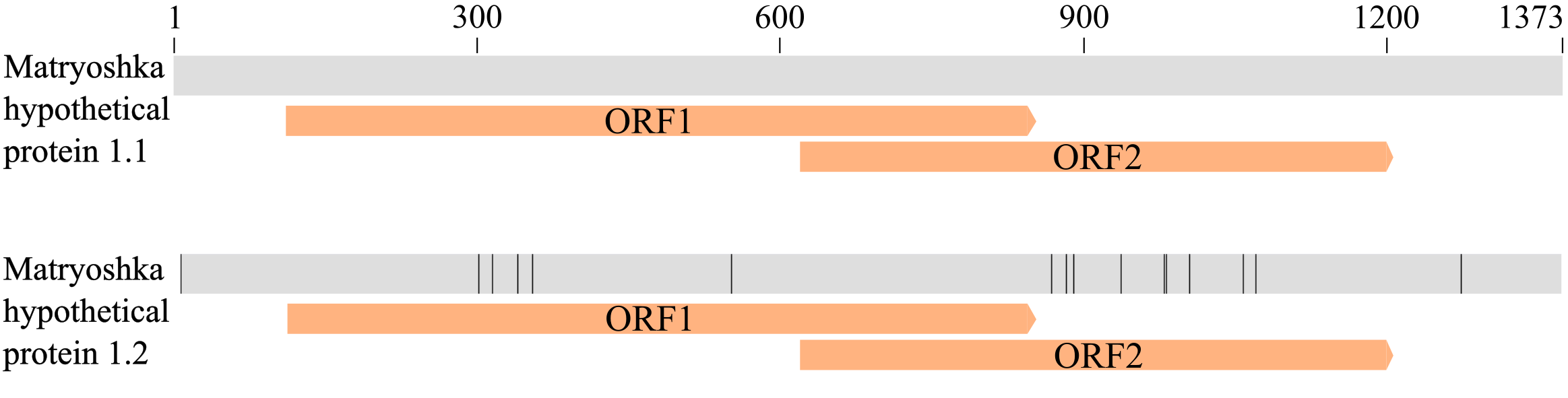

Supplement: S1 Fig — Nucleotide polymorphisms are indicated in black. (TIFF) [file pone.0319395.s003.tiff]
